# Interleukin-36γ is causative for liver damage upon infection with Rift Valley fever virus in type I interferon receptor-deficient mice

**DOI:** 10.3389/fimmu.2023.1194733

**Published:** 2023-09-01

**Authors:** Martina Anzaghe, Marc A. Niles, Eugenia Korotkova, Monica Dominguez, Stefanie Kronhart, Samira Ortega Iannazzo, Ingo Bechmann, Malte Bachmann, Heiko Mühl, Georg Kochs, Zoe Waibler

**Affiliations:** ^1^ Division of Immunology, Paul-Ehrlich-Institut, Langen, Germany; ^2^ Medical Faculty, Institute for Anatomy, University Leipzig, Leipzig, Germany; ^3^ Pharmazentrum Frankfurt/ZAFES, University Hospital Frankfurt, Goethe-University Frankfurt am Main, Frankfurt am Main, Germany; ^4^ Institute of Virology, Medical Center, Faculty of Medicine, University of Freiburg, Freiburg, Germany

**Keywords:** rift valley fever virus, type I interferon, interleukin-36γ, anti-inflammatory, immune pathology, dysregulation, liver injury

## Abstract

Type I interferons (IFN) are pro-inflammatory cytokines which can also exert anti-inflammatory effects via the regulation of interleukin (IL)-1 family members. Several studies showed that interferon receptor (IFNAR)-deficient mice develop severe liver damage upon treatment with artificial agonists such as acetaminophen or polyinosinic:polycytidylic acid. In order to investigate if these mechanisms also play a role in an acute viral infection, experiments with the *Bunyaviridae* family member Rift Valley fever virus (RVFV) were performed. Upon RVFV clone (cl)13 infection, IFNAR-deficient mice develop a severe liver injury as indicated by high activity of serum alanine aminotransferase (ALT) and histological analyses. Infected IFNAR^-/-^ mice expressed high amounts of IL-36γ within the liver, which was not observed in infected wildtype (WT) animals. In line with this, treatment of WT mice with recombinant IL-36γ induced ALT activity. Furthermore, administration of an IL-36 receptor antagonist prior to infection prevented the formation of liver injury in IFNAR^-/-^ mice, indicating that IL-36γ is causative for the observed liver damage. Mice deficient for adaptor molecules of certain pattern recognition receptors indicated that IL-36γ induction was dependent on mitochondrial antiviral-signaling protein and the retinoic acid-inducible gene-I-like receptor. Consequently, cell type-specific IFNAR knockouts revealed that type I IFN signaling in myeloid cells is critical in order to prevent IL-36γ expression and liver injury upon viral infection. Our data demonstrate an anti-inflammatory role of type I IFN in a model for virus-induced hepatitis by preventing the expression of the novel IL-1 family member IL-36γ.

## 1 Introduction

Type I interferons (IFN) are a family of cytokines which are expressed early upon infection in order to guarantee the survival of the host until the adaptive immunity is activated. In order to elicit their pro-inflammatory function, type I IFN are induced e.g. upon sensing of pathogen-associated molecular patterns (PAMPs). PAMPs are recognized by pattern recognition receptors (PRRs) such as toll-like receptors (TLR) and the retinoic acid-inducible gene (RIG)-I-like receptors (RLR). Whereas most TLR signal via the adaptor protein myeloid differentiation primary response 88 (MyD88), signaling of RLR is dependent on the adaptor protein mitochondrial antiviral signaling protein (MAVS) (1). Type I IFN can be subdivided into 13 isoforms of IFN-α, one IFN-β, as well as IFN-ϵ, IFN-τ, IFN-κ, IFN-ω, IFN-δ, IFN-ζ, and IFN-v (2). All type I IFN bind to a common type I IFN receptor (IFNAR) in order to regulate the expression of hundreds of IFN-stimulated genes (ISG) (2, 3). It has been shown by several studies in the past that a positive feedback loop via the IFNAR is necessary for the formation of robust type I IFN responses. Small amounts of early type I IFN, mainly IFN-α_4_ and IFN-β, bind to the IFNAR in order to induce the production of high amounts of type I IFN (4–6). Nevertheless, depending on dose and route of infection, the IFNAR feedback loop is not strictly necessary for robust type I IFN expression upon infection with a variety of RNA-encoded viruses (7).

Even though type I IFN are generally considered as pro-inflammatory cytokines, they can also exert anti-inflammatory functions. Impaired induction of type I IFN in a model of TLR9 ligand-induced liver injury resulted in increased inflammation and liver damage. Here, the protective role of type I IFN was mediated via the attenuation of IL-1β expression and induction of the IL-1 receptor antagonist (RA) (8). Moreover, IL-1 family member-mediated immune pathology was shown for alcohol-induced hepatitis, fatty liver disease, as well as in a mouse model investigating lipopolysaccharide (LPS)/d-galactosamine (D-GalN)-induced liver injury (9, 10). In addition, in a previous study we showed that the artificial double-stranded RNA polyinosinic:polycytidylic acid (poly(I:C)) induced severe liver injury in IFNAR-deficient mice (IFNAR^-/-^). Deficient type I IFN signaling was associated with increased levels of IL-1β, which in turn was causative for the induction of severe liver damage as shown e.g. by high activity of serum alanine aminotransferase (ALT) (11, 12). As many viruses produce double-stranded RNA during their life cycle, poly(I:C) resembles a good model for a viral infection. To further explore the anti-inflammatory role of type I IFN upon a ‘real’ acute viral infection, we aimed to investigate the regulation of IL-1 family members in virus-induced hepatitis.

Rift Valley fever is an emerging zoonotic disease endemic in sub-Saharan African countries and the Arabian Peninsula. The disease is caused by the Rift Valley fever virus (RVFV), which is an enveloped virus with a segmented negative-sensed single-stranded RNA genome. It belongs to the *Bunyaviridae* family, genus *Phlebovirus*, and is primarily transmitted via *Aedes mcintoshi* mosquitos (13–18). RVFV outbreaks in the human population vary in extent, intensity, and location. Thus far, the largest outbreak was documented in Egypt 1977 with 10,000-20,000 cases and a mortality rate of up to 20% (16). RVFV predominantly infects domestic ruminant animals including sheep, cattle, goats, and camels (16). In humans, infection with RVFV usually causes a self-limiting mild disease with influenza-like symptoms. Nevertheless, a small percentage of patients develop complications with clinical symptoms ranging from hemorrhagic fever to acute hepatitis, severe encephalitis, or thrombosis (13, 17, 19). The liver is considered the major target organ for RVFV and viral replication was shown to induce apoptosis in hepatocytes accompanied by necrosis (13, 15, 18). In line with that, RVFV pathogenesis in mice is associated with a loss of liver function due to liver necrosis and hepatitis (15, 16, 20).

RVFV was described to mainly infect epithelial cells (such as hepatocytes), mesenchymal cells, neural cells, hematopoietic cells such as mononuclear phagocytes, and cells morphologically consistent with dendritic cells (DC) (15). Other studies identified macrophages to be the primarily virus shedding cells while DC and granulocytes are additional target cells for RVFV replication (17).

The genome of RVFV consists of three segments: large (L), medium (M), and small (S) (16, 18). RVFV clone (cl)13 is an avirulent virus variant, which harbors a large deletion in the S segment. It is naturally attenuated and was obtained by plaque purification from a field isolate obtained from a nonfatal human case during an outbreak in 1974 (19). RVFV cl13 has no pathogenicity for mice or hamsters and the animals survive infections with up to 10^6^ plaque-forming units (pfu) without developing any signs of disease. Nevertheless, type I IFN are critical for the survival of mice upon RVFV cl13 infection as IFNAR^-/-^ mice show up to 100-fold higher titers when compared to their wildtype (WT) counterparts and finally succumb to infection (19).

In order to investigate if type I IFN also exert anti-inflammatory effects upon an acute viral infection, we used RVFV cl13 and analyzed the induction of IL-1 family members. Interestingly, data revealed a critical role for the novel IL-1 family member IL-36γ which was known thus far to play a pro-inflammatory role in some human disorders such as psoriasis, inflammatory bowel disease, pulmonary disease, or rheumatoid arthritis (21–24). IL-36 cytokines are new members of the IL-1 family which comprise IL-36α, IL-36β, and IL-36γ (25–27). All bind to a heterodimeric receptor composed of the IL-36 receptor (IL-36R) and IL-1 receptor accessory protein (IL-1RAcP). IL-36 receptor antagonist (IL-36RA) and IL-38 have been shown to negatively regulate the IL-36 signaling pathway via competitive binding to IL-36R, suppressing agonist recognition and IL-1RAcP recruitment (21, 28–30). IL-36R is expressed in skin (especially on keratinocytes), epithelial cells within the lung, the gastrointestinal tract, and other tissues. Furthermore, several types of immune cells were shown to express IL-36R and respond to stimulation. In addition, human and mouse DC express IL-36R and binding of IL-36 promotes DC maturation and improves antigen presentation via upregulation of HLA-DR, CD83, and CD86 (21, 29, 31–33). IL-36 stimulates the production of various cytokines, chemokines, adhesion molecules, and pro-inflammatory mediators. IL-36 cytokines can be induced upon stimulation with different agents including cytokines, TLR ligands, bacterial or viral infections, or other pathological conditions (30, 34, 35).

In this study, we provide for the first time a mechanism for the type I IFN-mediated regulation of IL-36γ induction in the context of an acute viral infection.

## 2 Materials and methods

### 2.1 Mice

C57BL/6 WT mice were purchased from Harlan Winkelmann (Borchen, Germany). IFNAR^-/-^ mice (6) were backcrossed at least 20 times on the C57BL/6 background. ISRE-eGFP mice express eGFP under the control of an interferon-stimulated response element (ISRE) (36). CD4Cre IFNAR^flox/flox^ and CD19Cre IFNAR^flox/flox^ mice (37), LysMCre IFNARflox/flox mice (38), and CD11cCre IFNARflox/flox (6) have been described before. To obtain MyD88^-/-^IFNAR^-/-^ double-deficient mice and MAVS^-/-^IFNAR^-/-^ double-deficient mice, MyD88^-/-^ mice or MAVS^-/-^ mice were intercrossed with IFNAR^-/-^ mice as described before (7). All mice were bred under specific pathogen free conditions at the Zentrale Tierhaltung of the Paul-Ehrlich-Institut. Correct gene knock outs were verified by PCR analyses for all genotypes used. Health monitoring results of sentinel mice for all knock out mice showed no differences when compared to WT mice in our breeding. Fur structure, bearing, nutrition, and mating behavior of all knock out mice were inconspicuous. Mouse experimental work was carried out using 8 to 12 week old mice in compliance with regulations of German animal welfare.

### 2.2 Viruses and stimuli

For infection experiments RVFV cl13 (19) was used (under BSL2 conditions). Virus was propagated and titrated on Vero cells. Virus supernatants were harvested without further purification. Plaque assay analyses were performed as described earlier (39). *In vivo* infections were performed with 2x10^4^ pfu RVFV cl13 in 200 µl via the intraperitoneal (i.p.) route. Recombinant human IL-1RA (Anakinra, kindly provided by Swedish Orphan Biovitrum) was diluted in PBS and i.p. injected 6 hours before, simultaneously with, and 10 hours after infection with 100 µg/g bodyweight in a maximal volume of 200 µl. Recombinant mouse IL-36γ/IL-1F9 (aa 13-164; R&D) was diluted in PBS and intravenously (i.v.) injected (1 µg in a volume of 200 µl). Recombinant mouse IL-36RA (R&D) was diluted in PBS and i.v. injected 12 hours and 24 hours hours after infection (6 µg in a volume of 200 µl).

### 2.3 Quantification of cytokine production and ALT activity

To determine serum cytokine levels and ALT activity, peripheral blood was taken retro-orbitally upon anesthetization using Isofluran (CP-Pharma) and serum was prepared. IL-36γ was determined by enzyme-linked immunosorbent assay (ELISA) according to manufacturer’s instructions (Cloud-Clone Corp). Levels of eight different cytokines were measured via ProcartaPlex multiplex immunoassay kit (Thermo Fisher Scientific) according to manufacturer’s instructions. ALT activity was determined using a commercially available kit (Hiss Diagnostics GmbH).

### 2.4 Histology

For histological analysis, livers were fixed in 10% buffered formalin and embedded in paraffin. Sections were stained with hematoxylin and eosin (H&E) as described before (11) and examined by light microscopy.

### 2.5 Quantitative real-time PCR

Total RNA was prepared from liver, spleen, and peritoneal exudate cells (PEC) using Trizol (Invitrogen)/chloroform extraction. Isolation of liver and spleen was described earlier (11). Samples were treated with DNaseI (Roche) for 15 min at 37°C. Absence of genomic DNA contamination was confirmed by standard PCR using a glyceraldehyde 3-phosphate dehydrogenase (GAPDH)-specific primer pair. Target- and reference-mRNA levels were examined by qRT-PCR using QuantiFast SYBR Green PCR kit (Qiagen). Primer pairs used were the following: GAPDH forward 5’-ACCACAGTCCATGCCATCAC-3’, GAPDH reverse 5’-TCCACCACCCTGTTGCTGTA-3’, pro-IL-1β forward 5’-TCTTTGAAGTTGACGGACCC-3’, pro-IL-1β reverse 5’-TGAGTGATACTGCCTGCCTG-3’, IL-1RA forward 5’-TCAGATCTGCACTCAATGCC-3’, IL-1RA reverse 5’-CTGGTGTTTGACCTGGGAGT-3’, IL-36γ forward 5’-CAGGCCCTTGTGACAGTTCCA-3’, IL-36γ reverse 5’- TTAGCAGCAAAGTAGGGTGTCCATTA-3’, IL-36α forward 5’- CCGATGAGCTGCCTGTTCTGC -3’, IL-36α reverse 5’- GTGGGCAGCTCCCTTTAGAGC -3’, IL-36β forward 5’- AATGTCAAGCCTGTCATTCTTAGC -3’, IL-36β reverse 5’- GTGGGCAGCTCCCTTTAGAGC -3’, IL-36RA forward 5’- CGCAGAGAAGGTCATTAAAGG -3’, IL-36RA reverse 5’- AGCTCTTTGATTCCTTGGC -3’. The expression levels of all target genes were normalized against GAPDH (ΔCt). Gene expression values were calculated based on the ΔΔCt method using the mean of the untreated control group as calibrator to which all other samples were compared. Relative quantities (RQ) were determined using the equation RQ=2^-ΔΔCt^.

### 2.6 Flow cytometry

Isolation of PEC was described elsewhere (7, 11). For FACS analyses, cells were stained for 20 min at 4°C with the following fluorochrome-labeled monoclonal antibodies: anti-CD11c-allophycocyanin (APC), anti-B220-PE (both from BD PharMingen), anti-CD11b-Pacific Blue (from Caltag/Invitrogen), and anti-F4/80-APC (AbD Serotec). Cells were washed and analyzed with a LSRII flow cytometer (Becton Dickinson). Analyses were performed using BD FACSDiva™ 8.0.1 and FlowJo® 7.6.5 and 10.7.1.

### 2.7 Statistics

For all animal experiments, the statistical evaluation was exploratory. All observed effects were described by specifying key statistical metrics (mean value, standard deviation, etc.). For normally distributed data, 95% confidence intervals are given for the mean estimates (also for mean differences). Statistical tests were decided at the two-sided significance level α=5%. For paired comparisons between several treatment groups, the associated p-values were adjusted. Data that are not normally distributed were transformed accordingly. If a transformation is not possible, non-parametric methods were used. All animal experiments were approved by the Regierungspräsidium Darmstadt with the license number F107_1027.

Either Welch’s t-test or Mann Whitney test were performed using GraphPad Prism 9.2.0.Values with p ≤ 0.05 are statistically significant which is illustrated by the number of stars: (*) for p ≤ 0.05, (**) for p ≤ 0.01, (***) and for p ≤ 0.001; ns = not significant.

## 3 Results

### 3.1 IFNAR^-/-^ mice develop severe liver damage upon infection with RVFV

**Figure 1:**
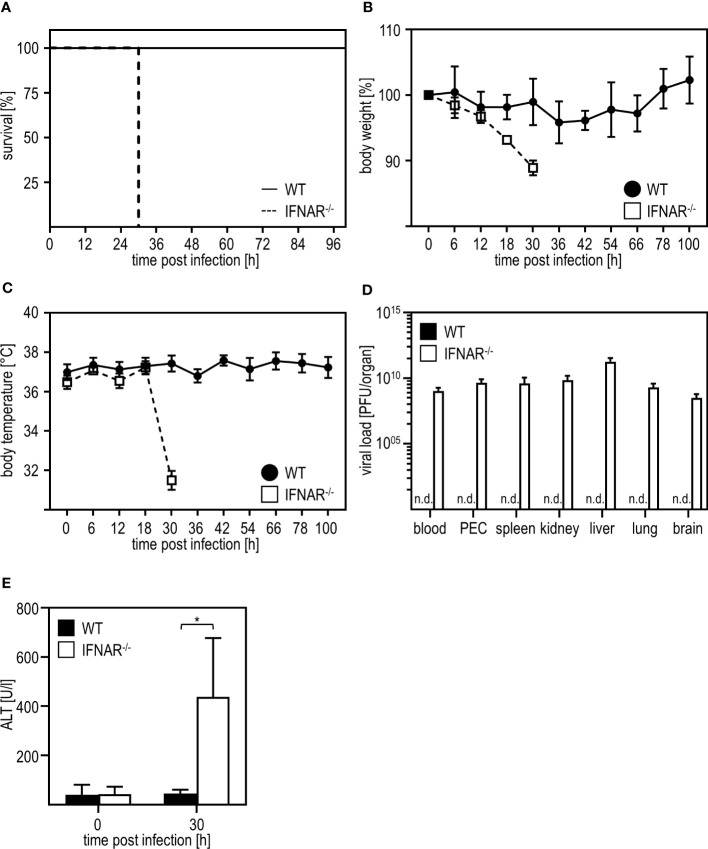
Infection with RVFV cl13 induces liver damage in type I IFN-deficient mice. C57BL/6 (WT) and IFNAR^-/-^ mice were i.p. infected with 2x10^4^ pfu/200 µl RVFV cl13 (n=4-7) and **(A)** survival, **(B)** body weight, and **(C)** body temperature was monitored at the indicated time points post infection. **(D)** Viral loads were analyzed 30 hours post infection (hpi) in the indicated organs by plaque assay (n=4). **(E)** ALT activity was measured 30 hpi within the serum of WT and IFNAR^-/-^ mice (n=6). Error bars indicate standard deviations. * < 0.05 (Welch’s t-test); n.d., not detectable.

As shown before (7), IFNAR^-/-^ mice succumb to infection with RVFV cl13, which is accompanied by body weight loss and a drop in body temperature, while their WT counterparts survive the infection and do not show such symptoms (**Figures 1A-C**). In line with this, WT mice mount IFN-α responses 12 hpi while IFNAR^-/-^ mice produced high amounts of IFN-α (up to ~4500 pg/ml) at 30 hpi indicating uncontrolled IFN-α production in the course of infection (**Supplementary Figure 1**). As given in **Figure 1D**, infection with RVFV induces viremia with high viral loads in all organs tested in IFNAR^-/-^ but not WT mice resulting in the death of IFNAR^-/-^ mice 30 hours post infection (7). In addition, IFNAR^-/-^ but not WT mice show high ALT activity in the peripheral blood 30 h post infection indicating a severe liver injury in these animals (**Figure 1E**). In conclusion, IFNAR^-/-^ but not WT mice develop a severe liver damage upon infection with RVFV cl13.

### 3.2 Liver damage in RVFV-infected IFNAR^-/-^ mice is caused by IL-1 family member IL-36γ

**Figure 2:**
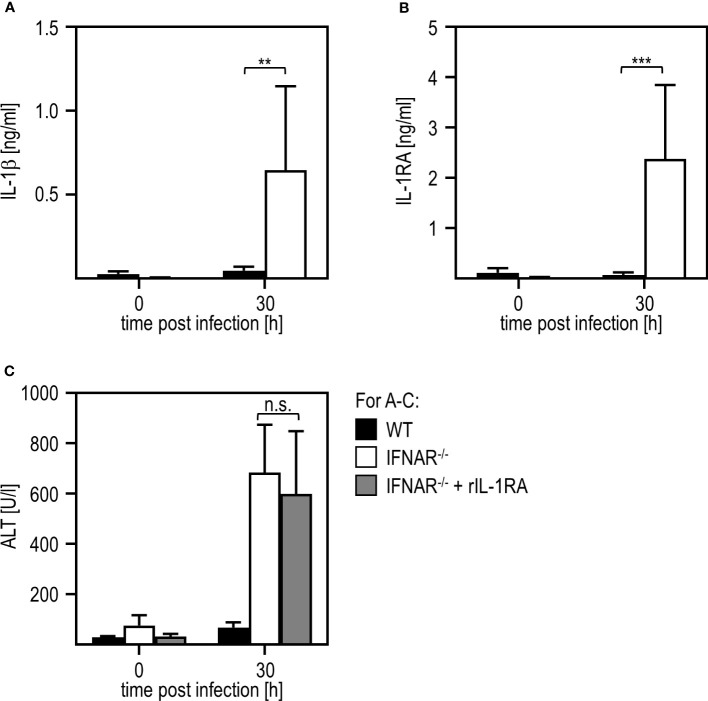
Liver injury in RVFV cl13-infected IFNAR^-/-^ mice is not mediated by a dysregulation of IL-1β/IL-1RA. C57BL/6 (WT) and IFNAR^-/-^ mice were i.p. infected with 2x10^4^ pfu/200 µl RVFV cl13. Levels of IL-1β **(A)** and IL-1RA **(B)** were measured 30 hpi within the serum by an ELISA method (n=4-13). **(C)** WT and IFNAR^-/-^ mice were infected with 2x10^4^ pfu/200 µl RVFV cl13. Then, a subset of infected IFNAR^-/-^ animals was treated with 100 µg recombinant IL-1RA (Anakinra) per g body weight as described in material methods. ALT activity was measured 30 hpi within the serum (n=4-13) Error bars indicate standard deviations. ** < 0.01; *** < 0.001 (Welch’s t-test); n.s., not significant.

Next, we aimed to investigate if liver damage upon RVFV cl13 infection is mediated by a dysregulation of the IL-1 family members IL-1β and/or IL-1RA as it has been shown before for poly(I:C) treatment (11, 12). Thus, we isolated livers of IFNAR^-/-^ and WT mice 30 hours post RVFV cl13 infection and analyzed IL-1β (**Figure 2A**) and IL-1RA (**Figure 2B**) induction by ELISA. Unlike poly(I:C)-treated animals (11, 12), RVFV cl13-infected IFNAR^-/-^ mice showed high IL-1β as well as IL-1RA expression while WT mice did not show any IL-1β or IL-1RA induction upon infection. To confirm that an IL-1β/IL-1RA imbalance is indeed not involved in RVFV cl13-mediated liver injury, RVFV cl13-infected IFNAR^-/-^ mice were treated with recombinant IL-1RA (Anakinra) and analyzed for the development of liver damage by analyzing ALT activity within the serum (**Figure 2C**). Here, the administration of recombinant IL-1RA could not prevent the development of severe liver injury in RVFV cl13-infected IFNAR^-/-^ mice indicating that IL-1β/IL-1RA are not involved in RVFV cl13-induced liver injury.

**Figure 3:**
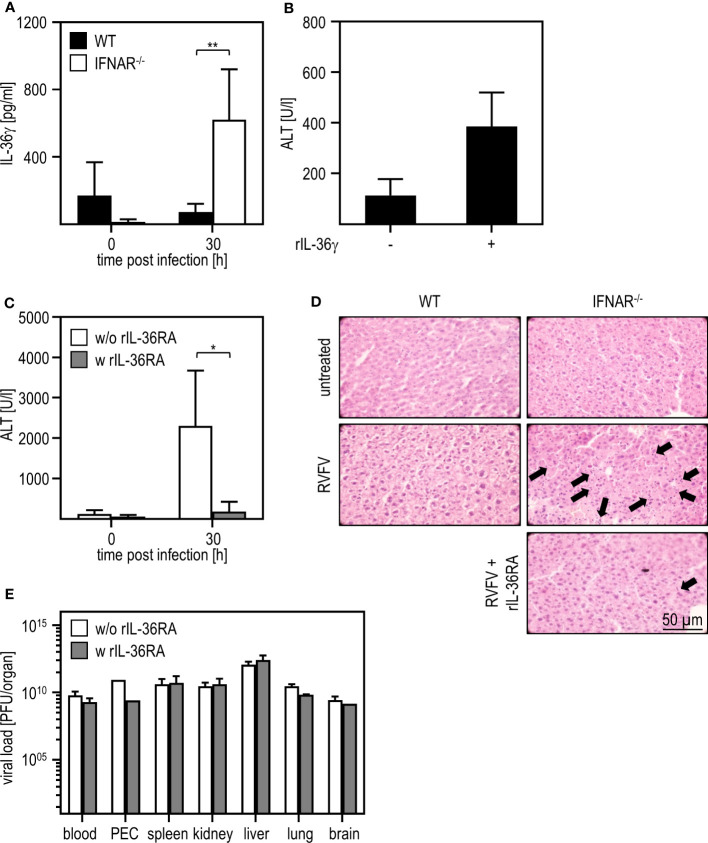
IL-36γ is causative for the liver injury in RVFV cl13-infected IFNAR^-/-^ mice. C57BL/6 (WT) and IFNAR^-/-^ mice were i.p. infected with 2x10^4^ pfu/200 µl RVFV cl13. **(A)** IL-36γ was measured 30 hpi within the serum using an ELISA method (n=4-6). **(B)** C57BL/6 (WT) mice were i.v. injected with 1 µg recombinant IL-36γ. ALT activity within the serum was measured 3 hours post treatment (n=3). **(C)** RVFV cl13-infected IFNAR^-/-^ mice were i.v injected with 6 µg rIL-36RA in 200 µl 12 and 24 hpi. RVFV cl13-infected IFNAR^-/-^ mice served as control. ALT activity was measured 30 hpi (n=5). **(D)** Histological analyses were performed using H&E staining. Liver sections of RVFV cl13-infected WT and IFNAR^-/-^ mice were prepared 30 hpi as described earlier (11). Additionally, RVFV cl13-infected IFNAR^-/-^ mice were rIL-36RA-treated (all n=2). Untreated animals served as controls. Arrows exemplarily indicate apoptotic bodies within the tissue. **(E)** Organs of WT and IFNAR^-/-^ mice were harvested 30 hours post RVFV infection and analyzed for the viral load by plaque assay (n=2-6). Error bars indicate standard deviations. * < 0.05; ** < 0.01 (Welch’s t-test).

In a model of paracetamol (acetaminophen, APAP)-induced liver damage, the IL-1 family member IL-36γ and its antagonist IL-36RA were shown to play a role in the onset and regeneration of liver damage (40). Hence, we presumed that these IL-1 family members might be type I IFN regulated and thus involved in the RVFV cl13-induced liver damage in absence of IFNAR-signaling. To obtain a first insight, we analyzed the expression of IL-36γ upon RVFV cl13 infection by an ELISA method. As given in **Figure 3A**, IFNAR^-/-^ but not WT mice show high levels of IL-36γ 30 hours post infection. To investigate if IL-36γ is sufficient to cause liver injury, we i.p. injected WT animals with 1 µg recombinant IL-36γ and analyzed ALT activity (**Figure 3B**). Indeed, WT mice developed a severe liver injury within 3 hours post IL-36γ injection as indicated by high ALT activity within the serum.

To further verify that IL-36γ was causative for the liver damage upon RVFV cl13 infection, RVFV cl13-infected IFNAR^-/-^ mice were treated with recombinant IL-36RA 12 and 24 hours post infection (**Figure 3C**). Analyses of IFNAR^-/-^ mice revealed that the administration of recombinant IL-36RA significantly reduced ALT activity (**Figure 3C**) and overall liver damage as indicated by histological analyses (**Figure 3D**). Of note, viral titers within all organs analyzed were not affected by IL-36RA treatment (**Figure 3E**). These data strongly indicate that the dysregulated IL-36γ expression in absence of type I IFN-signaling is causative for the liver injury observed upon RVFV cl13 infection.

### 3.3 Macrophage-like cells within the peritoneum are the main source of IL-36 γ in the absence of type I IFN signaling

**Figure 4:**
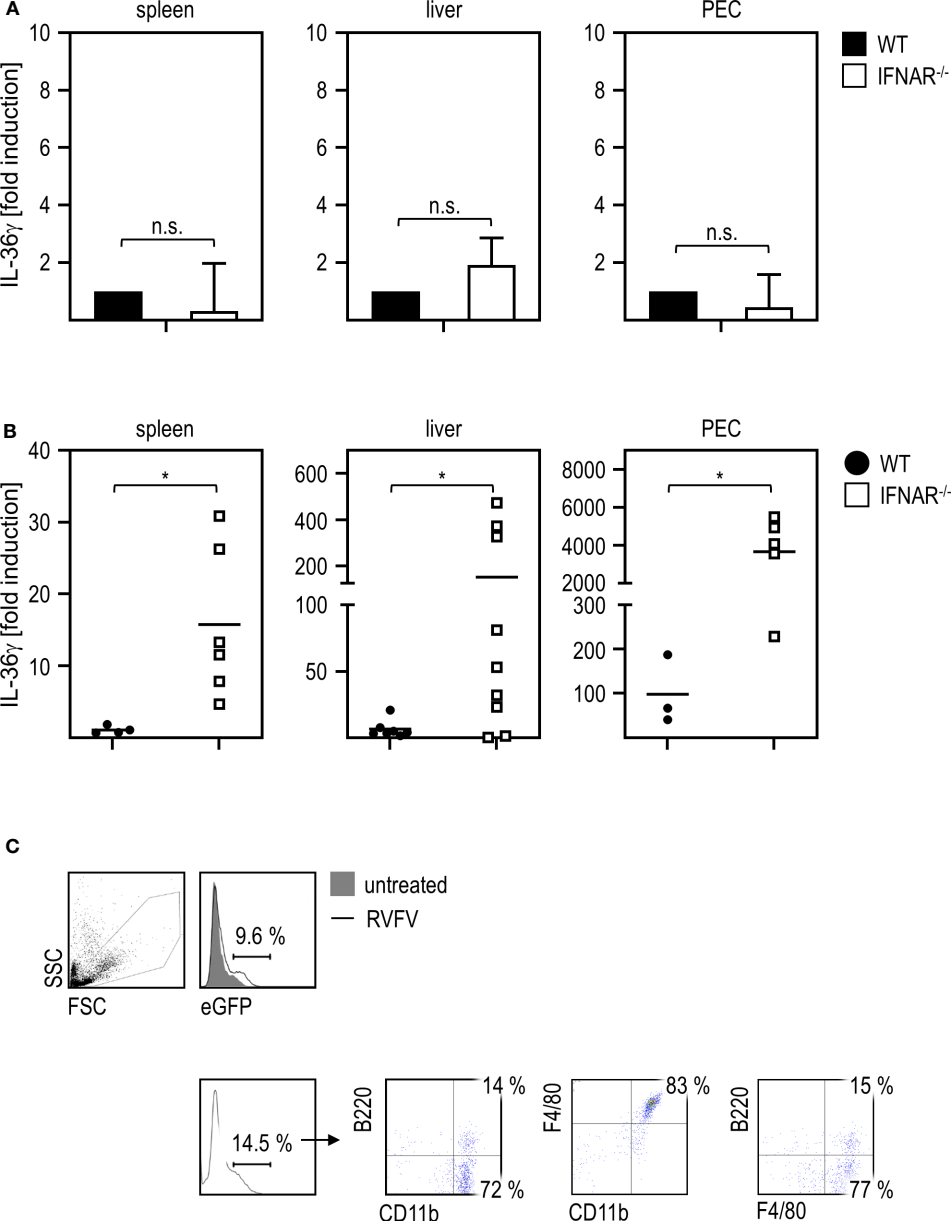
PEC are the main source of IL-36γ upon RVFV cl13 infection of IFNAR^-/-^ mice. **(A)** To exclude genotype-specific differences in basal expression levels, spleen, liver, and peritoneal exudate cells (PEC) of C57BL/6 (WT) and IFNAR^-/-^ mice (each n=4) were isolated and RNA was prepared as described elsewhere (11). Expression of IL-36γ was determined by qRT-PCR analyses. All values were normalized to WT animals; n.s.=not significant (Mann Whitney test). **(B)** C57BL/6 (WT) and IFNAR^-/-^ mice (n=4-9) were i.p. infected with 2x10^4^ pfu RVFV cl13 in 200 µl. Spleen, liver, and PEC were isolated 24 hpi infection and RNA was prepared as described earlier (11). Expression of IL-36γ was determined by qRT-PCR analyses. **(C)** ISRE-eGFP mice were either left untreated or infected with RVFV cl13 for 30 hours. PEC were isolated and analyzed for eGFP expression by flow cytometry. eGFP-positive cells were further characterized as CD11b^+^F4/80^+^ (one representative staining out of three is shown). Error bars indicate standard deviations; * < 0.05 (Welch’s t-test); n.s., not significant.

To gain insight in which cells or organs are the source of IL-36γ upon RVFV cl13 infection, we analyzed spleens (as an organ harboring many immune cells), livers (as the site where the damage occurs), and PEC (because of the intraperitoneal route of infection) of WT and IFNAR^-/-^ mice. To ensure that both genotypes show a comparable basal expression of IL-36γ, we analyzed uninfected mice by qRT-PCR analysis. As given in **Figure 4A**, spleen, liver, and PEC of WT and IFNAR^-/-^ mice showed comparable basal levels of IL-36γ mRNA. Next, we infected WT and IFNAR^-/-^ mice with RVFV for 24 hours and isolated spleen, liver, and PEC RNA for qRT-PCR analyses. As shown in **Figure 4B**, IFNAR^-/-^ mice express high levels of IL-36γ mRNA in all organs/cells tested with the highest expression in PEC (up to 4000-fold induction) while WT mice did not show upregulation in spleen and liver and only minor upregulation (up to 80-fold) in PEC. These results strongly indicate that PEC are the main source of IL-36γ in IFNAR^-/-^ mice upon RVFV cl13 infection and that type I IFN signaling in these cells prevents IL-36γ induction.

In order to clarify which cells within the peritoneum directly sense type I IFN upon infection, we used reporter mice expressing eGFP under the control of the ISRE for further experiments. Flow cytometric analyses of PEC derived from RVFV-infected mice demonstrated that eGFP-positive cells are mainly CD11b^+^F4/80^+^ and therefore show a myeloid/macrophage-like phenotype (**Figure 4C**).

**Figure 5:**
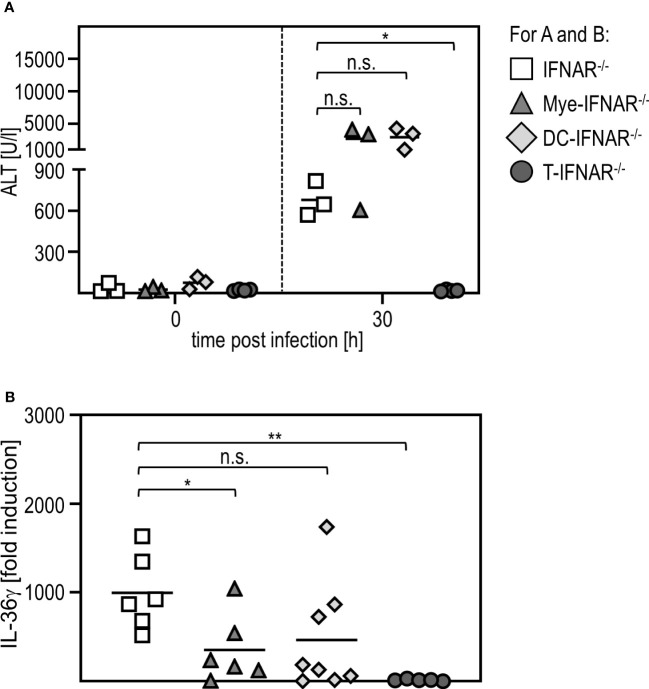
Myeloid cells need to sense type I IFN in order to protect from IL-36γ-mediated liver injury. IFNAR^-/-^, Mye-IFNAR^-/-^, DC-IFNAR^-/-^, and T-IFNAR^-/-^ mice were i.p. infected with 2x10^4^ pfu RVFV cl13 in 200 µl. **(A)** ALT activity was measured 0 and 30 hpi infection (n=3-4). **(B)** Induction of IL-36γ within the PEC was determined by qRT-PCR analyses (n=5-8); * < 0.05; ** < 0.01 (Welch’s t-test); n.s., not significant.

To investigate if sensing of type I IFN in myeloid/macrophage-like cells is critical for IL-36γ expression, we used conditional knockout mice, deficient for the IFNAR in certain types of immune cells (DC-IFNAR^-/-^ with specific IFNAR deletion in DC, Mye-IFNAR^-/-^ with specific IFNAR deletion in myeloid cells, T-IFNAR^-/-^ with specific IFNAR deletion in T cells, whereas all other cell types remain IFNAR-competent as described in (11)) and infected these mice with RVFV cl13. IFNAR^-/-^ mice served as control. As given in **Figure 5A**, mice deficient for the IFNAR in myeloid cells (Mye-IFNAR^-/-^) and DC (DC-IFNAR^-/-^) develop a severe liver injury upon infection which was even more pronounced when compared to IFNAR^-/-^ mice. In contrast, T-IFNAR^-/-^ control-mice did not show enhanced ALT activity upon infection. In line with this, qRT-PCR analyses of PEC derived from those animals show IL-36γ induction in Mye-IFNAR^-/-^ (350-fold) and DC-IFNAR^-/-^ (500-fold) which was not observed in T-IFNAR^-/-^ mice. As shown before, IFNAR^-/-^ mice strongly upregulate IL-36γ mRNA upon infection (1000-fold) (**Figure 5B**). Of note, IL-36γ induction in Mye-IFNAR^-/-^ mice was comparable to IFNAR^-/-^ mice. These results indicate that upon RVFV cl13 infection the absence of IFNAR-signaling in myeloid cells/DC results in IL-36γ expression, which in turn mediates a severe liver damage.

### 3.4 MAVS is critically involved in the production of IL-36γ upon RVFV infection of IFNAR^-/-^ mice

**Figure 6:**
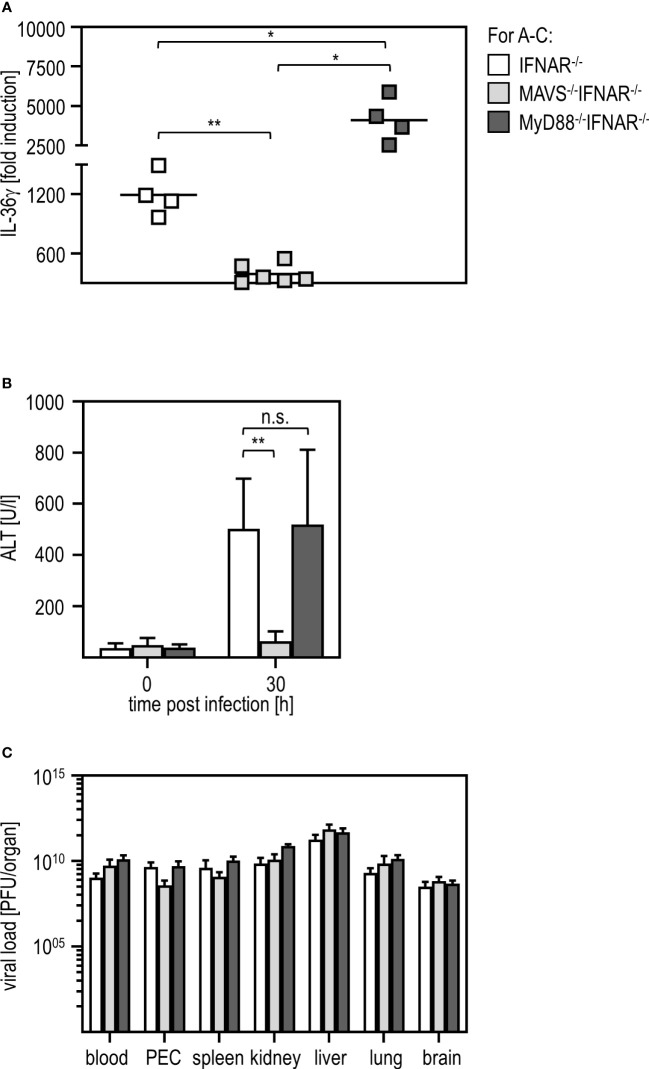
MAVS is critically involved in IL-36γ induction and IL-36γ-mediated liver injury upon RVFV cl13 infection of IFNAR^-/-^ mice. IFNAR^-/-^, MAVS^-/-^IFNAR^-/-^, and MyD88^-/-^IFNAR^-/-^ mice were i.p. infected with 2x10^4^ pfu RVFV cl13 in 200 µl for 30 hours. **(A)** IL-36γ induction was investigated by qRT-PCR analyses of the PEC (n=4-6). **(B)** ALT activity was measured in serum samples at 30 hours post infection (n=4-8). **(C)** Viral load in different organs was analyzed by plaque assay (n=4-7). Error bars indicate standard deviations; * < 0.05; ** < 0.01; n.s., not significant.

Viral infections can be sensed via different pathways such as those involving TLR or RLR (41, 42). To uncover which signaling pathway is involved in IL-36γ induction and liver damage upon RVFV infection, we used IFNAR^-/-^ mice additionally deficient for MyD88 (MyD88^-/-^IFNAR^-/-^ affecting most TLR pathways) and IFNAR^-/-^ mice additionally deficient for MAVS (MAVS^-/-^IFNAR^-/-^ not capable of using the RLR pathway). qRT-PCR analysis of PEC derived from those mice revealed that MAVS^-/-^IFNAR^-/-^ did not induce any IL-36γ mRNA upon RVFV cl13 infection while IFNAR^-/-^ and MyD88^-/-^IFNAR^-/-^ showed high levels of IL-36γ mRNA. Interestingly, IL-36γ mRNA-levels were even significantly higher in MyD88^-/-^IFNAR^-/-^ mice when compared to IFNAR^-/-^ mice (**Figure 6A**). These data show that for IL-36γ mRNA expression by IFNAR^-/-^ PEC, the MAVS-adapted RLR pathway is used while the MyD88-adapted TLR are of no relevance. Finally, we analyzed the ALT activity in the serum of these different knockout mice. While MyD88^-/-^IFNAR^-/-^ showed levels comparable to those in IFNAR^-/-^ mice, no ALT activity was detected in MAVS^-/-^IFNAR^-/-^ mice indicating that these mice were indeed protected from the RVFV cl13-induced IL-36γ-mediated liver injury. Of note, plaque assay analyses revealed high viral loads in all organs tested, irrespective of the genotype of mice investigated (**Figure 6C**). Of note, higher levels of IL-36γ expression in MyD88^-/-^IFNAR^-/-^ could not be correlated with increased viral loads in these mice.

In conclusion, our study demonstrated a critical role for the IL-1 family member IL-36γ for the induction of liver damage in the course of viral infection. In line with results obtained using the artificial double-stranded RNA poly(I:C), the dysregulation of IL-1 family members in the absence of type I IFN results in severe liver injury independent of viral titers.

## 4 Discussion

It was shown before that members of the IL-1 family, especially IL-1β, play an important role in liver injury (11, 43). The current study demonstrates a pathological role for IL-36γ upon an acute viral infection. The pro-inflammatory cytokine IL-36γ was known before to be critically involved in several inflammatory disorders such as psoriasis (29, 44, 45), inflammatory bowel disease (23, 32, 44), rheumatoid arthritis (21), or systemic lupus erythematosus (SLE) (24). Accumulating evidences suggest that IL-36γ also plays a role during infectious diseases (24). Nevertheless, it is not fully understood yet if it promotes infection, drives immune-pathology, or rather plays a protective role.

A study by Wang et al. using a mouse model for influenza A virus (IAV) infection revealed that IL-36 contributes to lung damage and mortality by promoting inflammation. Mice deficient for the IL-36R were protected from IAV-induced lung injury and mortality. Furthermore, IL-36R^-/-^ mice showed reduced lymphocyte activation, accumulation of myeloid cells, reduced permeability of the alveolar epithelial barrier, and less production of pro-inflammatory cytokines and chemokines (24, 46). In line with that, patients with IAV-induced acute respiratory syndrome (ARDS) show higher concentrations of IL-36γ in the plasma when compared to healthy individuals (47). In addition, IL-36γ and IL-36α are significantly upregulated in patients with pulmonary tuberculosis, bacterial pneumonia, or chronic hepatitis B virus (HBV) infection (47, 48).

Others reported a protective role for IL-36. Upon infection of mice, pretreatment with IL-36γ increased the resistance against Herpes simplex virus (HSV)-2 infection and disease (21). Mice lacking IL-36γ showed increased morbidity and mortality upon IAV infection which was associated with increased virus titers and higher levels of inflammatory cytokines (46). Furthermore, IL-36γ^-/-^ mice showed higher bacterial load in the lung, systemic dissemination, and higher mortality upon infection with *Staphylococcus pneumoniae* when compared to WT mice (45). Blocking IL-36γ receptor binding and using recombinant IL-36γ (see **Figure 3**), we found IL-36γ to be causative for the liver damage induced upon infection with RVFV cl13 in absence of intact type I IFN signaling. Interestingly, neither IL-36α, IL-36β, nor IL-36RA were elevated in organs of RVFV cl13-infected IFNAR^-/-^ mice when compared to untreated IFNAR^-/-^ or WT mice (see **Supplementary Figure 2**).

IL-36γ was shown before to play a role in drug-induced liver injury. In a model of acetaminophen (APAP)-induced hepatitis, Scheiermann et al. showed elevated levels of IL-36γ within the liver of IFNAR^-/-^ mice. However, application of IL-36RA increased the late phase of liver injury indicating a potential role for IL-36γ as a cytokine contributing to the decision between tissue damage and liver regeneration (40). Moreover, treatment with IL-36RA significantly reduced the production of pro-inflammatory cytokines in BALB/c mice in a model of Concanavalin (Con)A-induced liver injury (49). ConA-treated IL-36R^-/-^ animals exhibited exaggerated T cell responses as shown by increased infiltration of effector T cells into the liver accompanied by the production of pro-inflammatory cytokines (29).

Interestingly, in our study, IL-36γ-mediated liver injury upon RVFV infection was completely independent from viral loads in various organs tested. In particular, while viral titers in MAVS^-/-^IFNAR^-/-^ animals are comparable to those in IFNAR^-/-^ mice (**Figures 1**, **6**), ALT activity in these double-deficient mice is comparable to WT animals (**Figure 6**). This suggests an immunopathology caused by IL-36γ expression in an IFNAR-deficient situation, whereas the hepatocellular tropism of RVFV and thus a virus-induced hepatitis seem to play a minor role.

Upon drug-induced liver injury, hepatocytes were shown to be the major source of IL-36 promoting the inflammatory response (50). In line with this, IL-6, TNF-α, and IFN-γ were slightly elevated in RVFV cl13-infected IFNAR^-/-^ mice when compared to RVFV cl13-infected WT mice (for all p-value = 0.0571, Mann-Whitney-Test). In addition, a minor expression of IL-12p70 and IL-18 was observed in RVFV cl13-infected IFNAR^-/-^ mice while other cytokines such as IL2, IL-4 or IL-5 did not differ between RVFV cl13-infected IFNAR^-/-^ and WT mice (**Supplementary Figure 3**). Our study revealed that type I IFN sensing by macrophages as well as DC is critical for the prevention of IL-36γ production and thus the IL-36γ-induced liver injury upon RVFV cl13 infection (see **Figure 5**). The protective role of myeloid cells was also shown before for poly(I:C)-induced liver damage. Here, myeloid-derived suppressor cells infiltrated the liver in a type I IFN-dependent manner in order to produce IL-1RA and therefore prevent IL-1β-mediated liver injury (11). Interestingly, in the current study, no enhanced IL-36RA expression was detected in qRT-PCR analyses of organs of WT mice (see **Supplementary Figure 2**) indicating that other factors are involved in order to regulate the expression of IL-36γ. Within the poly(I:C)-induced model of liver damage as well as in our current study, myeloid cells within the peritoneum are of particular importance. Along this line, deficient type I IFN signaling was associated with decreased liver recruitment of DC in a model of TLR9 ligand-induced liver damage (8). In addition, a study by Pinto et al. demonstrated that deletion of the IFNAR on subsets of myeloid cells such as macrophages and DC, resulted in uncontrolled replication of West Nile virus (WNV), production of pro-inflammatory cytokines, organ damage, and death (51). Interestingly, particularly cells of the macrophage lineage show increased susceptibility to RVFV cl13 infection in the absence of type I IFN. During initial stages of infection of IFNAR^-/-^ mice, RVFV cl13 replicates within macrophages and DC (17). Ermler et al. demonstrated that murine conventional DC and macrophages express type I IFN in response to RVFV cl13 (14, 18).

In addition, RVFV cl13-infected human monocyte-derived macrophages were shown to secrete TNF-α and type I IFN (18). This together with our data implies that production as well as sensing of type I IFN by myeloid cells such as macrophages and DC, is critical in order to prevent IL-36γ production and thus the IL-36γ-induced liver damage upon RVFV cl13 infection. Besides immune cells, human lung fibroblasts and human bronchial epithelial cells were shown to produce IL-6 and CXCL8 upon treatment with IL-36 (30). Thus, other cells than immune cells such as hepatocytes might contribute to a production of pro-inflammatory cytokines and dysregulated immune reaction upon RVFV cl13 infection.

It was shown in several studies that myeloid cells sense PAMPs rather via RLRs than TLRs. For example, Dutta et al. revealed that MAVS signaling in myeloid cells was critical for the resistance to Ebola virus infection in mice (52). In line with this, a variety of RNA-encoded viruses were shown to induce IFNAR-independent type I IFN responses in a MAVS-dependent manner (7). Here, we show that IL-36γ production and accordingly liver damage were dependent on MAVS in RVFV cl13-infected IFNAR^-/-^ mice while MyD88-adapted TLR are of no importance for IL-36γ production (see **Figure 6**). Along this line, others showed that upon infection with WNV no cytokine production was observed in MAVS^-/-^IFNAR^-/-^ mice (51). Of note, production of pro-inflammatory cytokines by DC or fibroblasts upon IL-36γ stimulation was shown to be dependent on MyD88 indicating that induction and sensing of IL-36γ can be mediated by different pathways (23, 45).

The interaction between type I IFN and IL-36 was previously observed also in psoriasis patients. Here, the expression of IL-36 strongly correlated with type I IFN overexpression (53). In addition, IL-36 serum levels in patients correlate with SLE disease activity, a disorder characterized by an enhanced type I IFN signature (53). In epithelial cells, IL-36 was shown to increase antiviral immunity by enhancing the expression of type I IFN stimulated genes (ISG) via the IFNAR (54). This mechanism may have evolved in order to control viruses that developed immune evasion strategies by blocking the production of type I IFN.

In conclusion, our study demonstrated that pro-inflammatory IL-36γ is causative for the observed liver injury in IFNAR^-/-^ mice upon an acute viral infection. The expression of IL-36γ is regulated by type I IFN, which need to be sensed by myeloid cells in order to prevent liver damage.

## Data availability statement

The raw data supporting the conclusions of this article will be made available by the authors, without undue reservation.

## Ethics statement

The animal study was approved by Regierungspräsidium Darmstadt, license number F107-1027. The study was conducted in accordance with the local legislation and institutional requirements.

## Author contributions

All authors contributed either to research design (MA, ZW, and GK) and/or the acquisition (MA, MN, EK, MD, SK, IB, SO-I), data analysis (MA, ZW, MN, EK, MD, SK, IB, SO-I), or interpretation of data (all authors). MA and ZW drafted the manuscript, which was critically revised by all other authors. All authors contributed to the article and approved the submitted version.

## Supplementary material

Supplementary Figure 1 RVFV cl13-infected IFNAR^-/-^ mice express high levels of IFN-α in the serum 30 hpi while WT mice show low amounts 12 hpi. C57BL/6 (WT) and IFNAR^-/-^ mice (n= 2-14) were i.p. infected with 2x10^4^ pfu RVFV cl13 in 200 µl. Serum was collected prior to infection as well as 30 hpi and tested using a multiplex kit for the presence of IL-6, TNF-α, and IFN-γ as described in the material and methods section. Error bars indicate standard deviations; *< 0.05; ** < 0.01; **** < 0.0001 (Welch’s t-test); n.s., not significant.

Supplementary Figure 2 Spleen, liver, and PEC of RVFV cl13-infected IFNAR^-/-^ mice do not upregulate IL-36α, Il-36β, or IL-36RA when compared to WT mice. C57BL/6 (WT) and IFNAR^-/-^ mice (for IL-36α and IL-36β n=3-5; for IL-36RA n= 2-11) were i.p. infected with 2x10^4^ pfu RVFV cl13 in 200 µl. Spleen, liver, and PEC were isolated 24 hpi infection and RNA was prepared as described earlier (11). Expression of IL-36α, IL-36β, and IL-36RA was determined by qRT-PCR analyses. n.s., not significant (Welch’s t-test).

Supplementary Figure 3 RVFV cl13-infected IFNAR^-/-^ mice show slightly enhanced levels of IL-6, TNF-α, and IFN-γ within the serum when compared to WT mice. C57BL/6 (WT) and IFNAR^-/-^ mice (n= 3-4) were i.p. infected with 2x10^4^ pfu RVFV cl13 in 200 µl. Serum was collected prior to infection as well as 30 hpi and tested using a multiplex kit for the presence of a panel of eight different cytokines as described in the material and methods section. n.s., not significant (Mann-Whitney-Test).

## Glossary

**Table:**

ALT	alanine aminotransferase
APAP	acetaminophen
CD	cluster of differentiation
cl	clone
ConA	concanavalin A
DC	dendritic cell(s)
D-GalN	d-galactosamine
ELISA	enzyme-linked immunosorbent assay
Figure	figure(s)
GFP	green fluorescent protein
HBV	hepatits B virus
hpi	hours post infection
HSV	herpes simplex virus
IAV	influenza A virus
IFN	type I interferon(s)
IFNAR	type I interferon receptor
IL	interleukin
IL-1RA	interleukin-1 receptor antagonist
IL-1RAcP	interleukin-1 receptor accessory protein
IL-36RA	interleukin-36 receptor antagonist
i.p.	intraperitoneal
ISG	IFN-stimulated gene(s)
i.v.	intravenously
LPS	lipopolysaccharide
MAVS	mitochondrial antiviral-signaling protein
MyD88	myeloid differentiation primary response 88
RVFV	Rift Valey fever virus
PAMP	pathogen associated molecular pattern
PBMC	peripheral mononuclear cells
PEC	peritoneal exudate cells
poly(I:C)	polyinosinic-polycytidylic acid
PRR	pattern recognition receptor(s)
RIG	retinoic acid-inducible gene
RLR	retinoic acid-inducible gene RIG-I-like receptors
SLE	systemic lupus erythematosus
TLR	toll like receptor(s)
WT	wildtype
