# Supplementary figures and images for: Interleukin-36γ is causative for liver damage upon infection with Rift Valley fever virus in type I interferon receptor-deficient mice
Source: Front Immunol. 2023 Sep 1;14:1194733. doi: 10.3389/fimmu.2023.1194733 (PMC10502725; doi:10.3389/fimmu.2023.1194733)

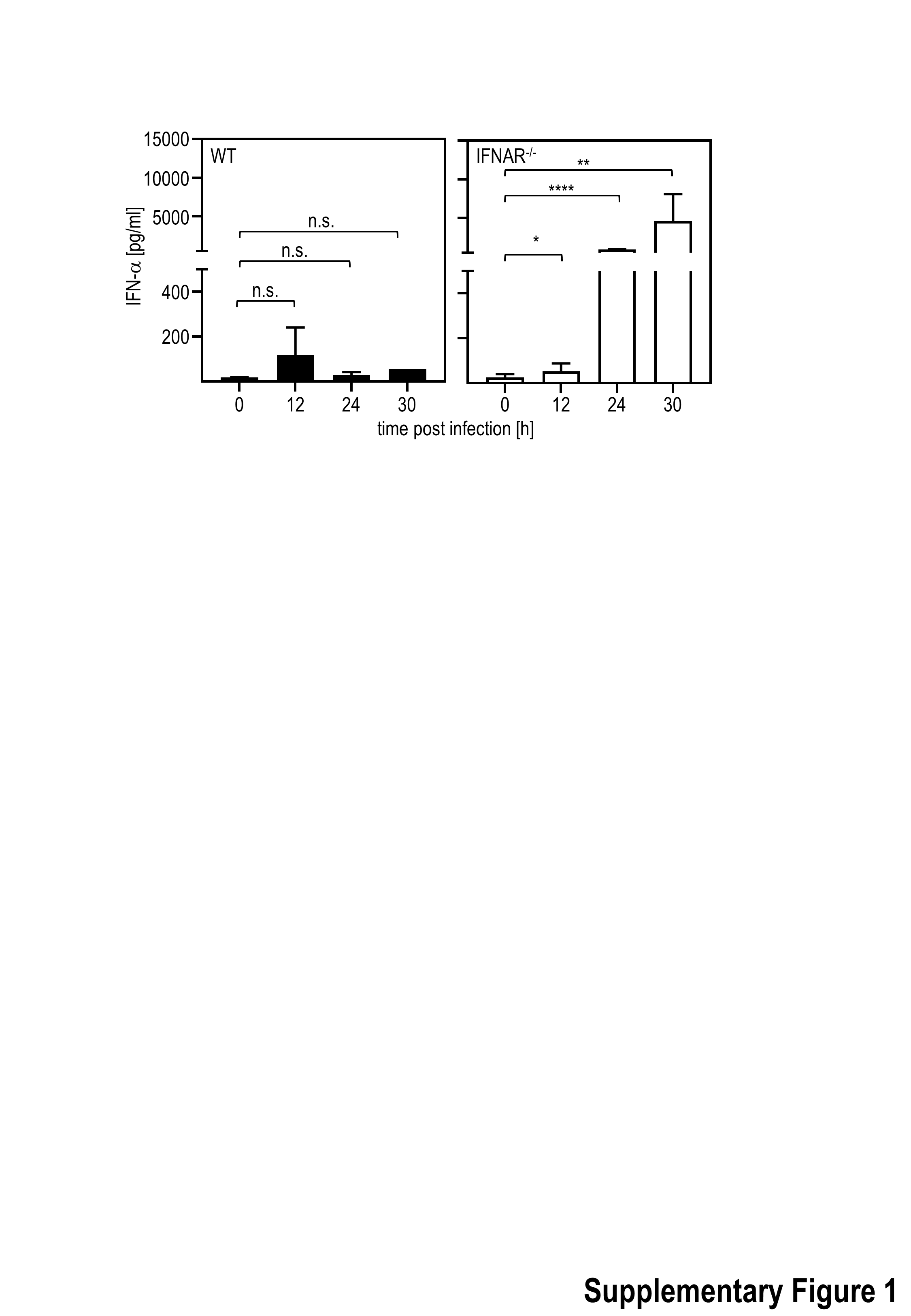

Supplement: Supplementary Figure 1 — RVFV cl13-infected IFNAR-/- mice express high levels of IFN-α in the serum 30 hpi while WT mice show low amounts 12 hpi. C57BL/6 (WT) and IFNAR-/- mice (n= 2-14) were i.p. infected with 2x10^4^ pfu RVFV cl13 in 200 µl. Serum was collected prior to infection as well as 30 hpi and tested using a multiplex kit for the presence of IL-6, TNF-α, and IFN-γ as described in the material and methods section. Error bars indicate standard deviations; *< 0.05; ** < 0.01; **** < 0.0001 (Welch’s t-test); n.s., not significant. [file Image_1.tif]

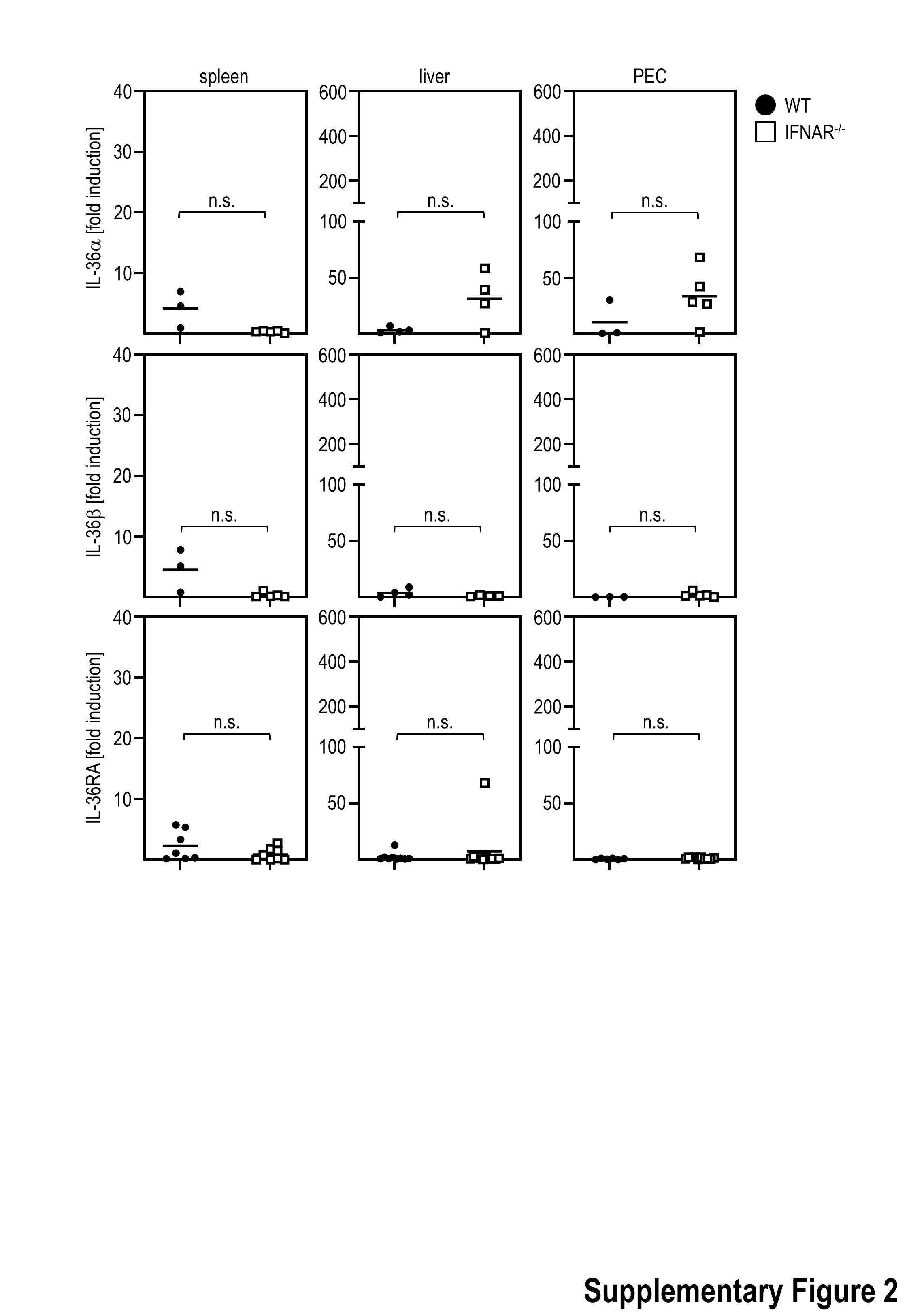

Supplement: Supplementary Figure 2 — Spleen, liver, and PEC of RVFV cl13-infected IFNAR-/- mice do not upregulate IL-36α, Il-36β, or IL-36RA when compared to WT mice. C57BL/6 (WT) and IFNAR-/- mice (for IL-36α and IL-36β n=3-5; for IL-36RA n= 2-11) were i.p. infected with 2x10^4^ pfu RVFV cl13 in 200 µl. Spleen, liver, and PEC were isolated 24 hpi infection and RNA was prepared as described earlier (11). Expression of IL-36α, IL-36β, and IL-36RA was determined by qRT-PCR analyses. n.s., not significant (Welch’s t-test). [file Image_2.tif]

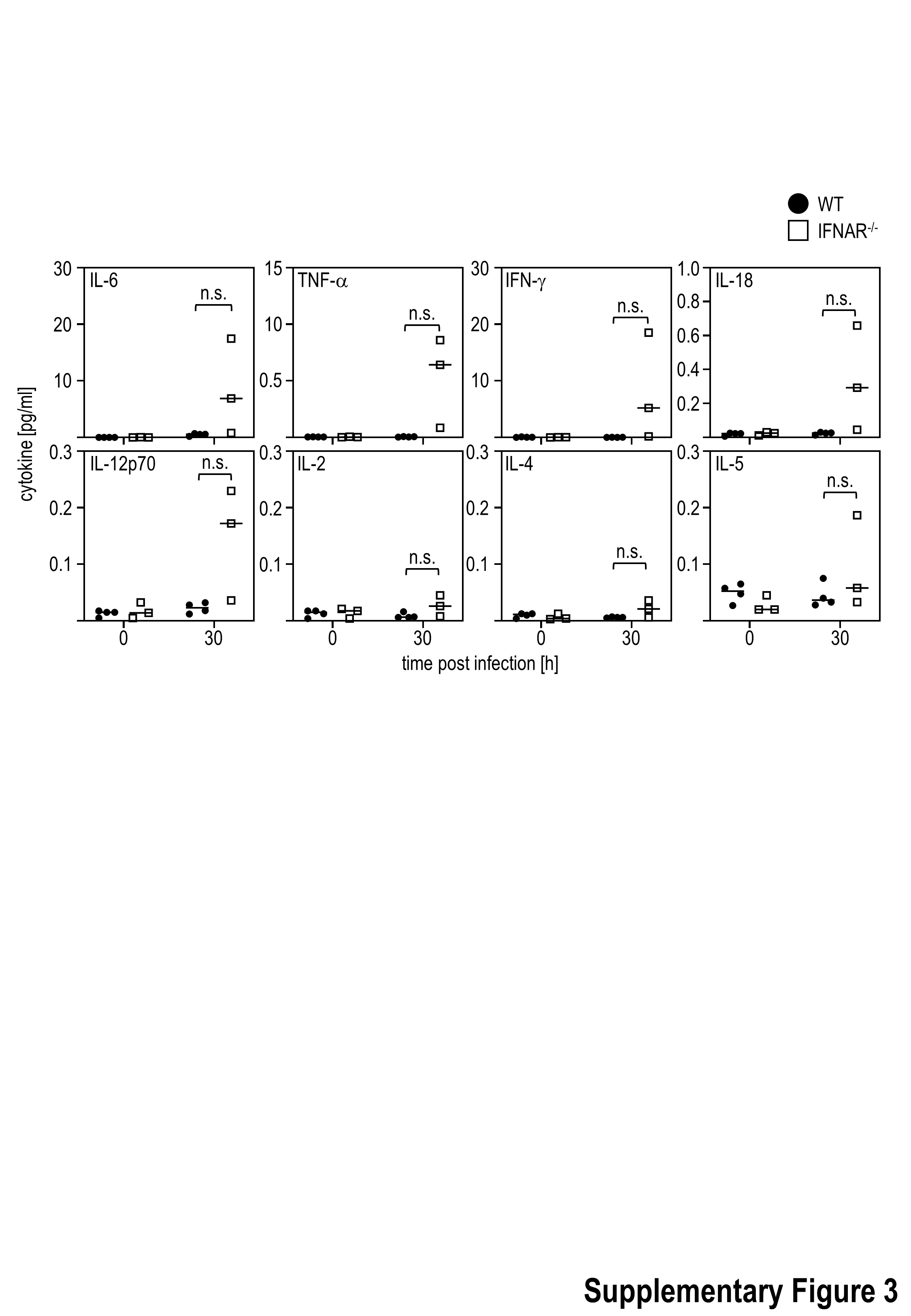

Supplement: Supplementary Figure 3 — RVFV cl13-infected IFNAR-/- mice show slightly enhanced levels of IL-6, TNF-α, and IFN-γ within the serum when compared to WT mice. C57BL/6 (WT) and IFNAR-/- mice (n= 3-4) were i.p. infected with 2x10^4^ pfu RVFV cl13 in 200 µl. Serum was collected prior to infection as well as 30 hpi and tested using a multiplex kit for the presence of a panel of eight different cytokines as described in the material and methods section. n.s., not significant (Mann-Whitney-Test). [file Image_3.tif]
